# Preclinical evaluation and preliminary clinical study of ^68^Ga-NODAGA-NM-01 for PET imaging of PD-L1 expression

**DOI:** 10.1186/s40644-025-00826-8

**Published:** 2025-01-27

**Authors:** Lingzhou Zhao, Jiali Gong, Sisi Liao, Wenhua Huang, Jinhua Zhao, Yan Xing

**Affiliations:** 1 Department of Nuclear Medicine, Shanghai General Hospital, Shanghai Jiao Tong University School of Medicine, Hongkou District, No. 100, Haining Road, Shanghai, 200080 China; 2 Nanomab Technology Limited, No. 333, North Chengdu Road, Jingan District, Shanghai, 200041 China

**Keywords:** Nanobody, Programmed death ligand-1, Non-small-cell lung cancer, PET imaging

## Abstract

**Background:**

Programmed cell death 1/programmed death ligand-1 (PD-L1)-based immune checkpoint blockade is an effective treatment approach for non-small-cell lung cancer (NSCLC). However, immunohistochemistry does not accurately or dynamically reflect PD-L1 expression owing to its spatiotemporal heterogeneity. Herein, we assessed the feasibility of using a ^68^Ga-labeled anti-PD-L1 nanobody, ^68^Ga-NODAGA-NM-01, for PET imaging of PD-L1.

**Methods:**

Micro-PET/CT and biodistribution studies were performed on PD-L1-positive and -negative tumor-bearing mice. Additionally, a preliminary clinical study was performed on two patients with NSCLC. NM-01 was radiolabeled with ^68^Ga without further purification under mild conditions.

**Results:**

^68^Ga-NODAGA-NM-01 exhibited radiochemical purity (> 98%), high stability *in vitro*, and rapid blood clearance *in vivo*. Specific accumulation of ^68^Ga-NODAGA-NM-01 was observed in PD-L1-positive tumor-bearing mice, with a good tumor-to-background ratio 0.5h post-injection. Furthermore, ^68^Ga-NODAGA-NM-01 PET/CT imaging was found to be safe with no adverse events and distinct uptake in primary and metastatic lesions of the PD-L1-positive patient, with a higher maximal standardized uptake value than that in lesions of the PD-L1-negative patient 1h post-injection.

**Conclusions:**

^68^Ga-NODAGA-NM-01 can be prepared using a simple method under mild conditions and reflect PD-L1 expression in primary and metastatic lesions. However, our findings need to be confirmed in a large cohort.

**Trial registration:**

NCT02978196. Registered February 15, 2018.

**Supplementary Information:**

The online version contains supplementary material available at 10.1186/s40644-025-00826-8.

## Background

Lung cancer is the leading cause of cancer-related mortality, with approximately 2.5 million new cases and 1.8 million deaths worldwide in 2022 [1–3]. In 2022, the National Cancer Center of China released annual cancer registration data, reporting 828,100 new cases and 657,000 deaths attributed to lung cancer nationwide in 2016. Non-small-cell lung cancer (NSCLC) constituted nearly 80–85% of all lung cancer cases [4]. Recently, programmed cell death 1 (PD-1)/programmed death ligand-1 (PD-L1) blockade immunotherapy has emerged as a highly promising approach for the comprehensive treatment of NSCLC. This strategy improves the durable response rate, providing long-lasting benefits for advanced NSCLC compared to conventional chemotherapy or radiotherapy [4–7].

PD-1 expression is upregulated in activated T cells, whereas its dominant ligand PD-L1 is primarily expressed on tumor cells following exposure to pro-inflammatory cytokines in the tumor microenvironment, resulting in adaptive immune resistance [6, 8]. High PD-L1 expression in patients with NSCLC correlates with shorter survival time and poorer prognosis [9]. To block tumor immune escape, immune checkpoint inhibitors are used to relieve the inhibitory effect of tumors on T lymphocytes and enhance the immune response. Consequently, they have become effective and safe therapeutic options for advanced NSCLC. Several PD-1/PD-L1 inhibitors, such as nivolumab, pembrolizumab, and atezolizumab have received clinical approval [10, 11]. Immunohistochemistry (IHC) is recommended for assessing PD-L1 expression in NSCLC. Considering the invasiveness of the procedures required for IHC (tissue collection), spatial and temporal heterogeneity, the impossibility of repeated and dynamic evaluation, and a higher risk of false-negative results, it is difficult for pathologists to provide precise measurements [12, 13]. Hence, there is an urgent need to design and develop a non-invasive and accurate imaging method to monitor PD-L1 expression for optimal personalized treatment.

Radiolabeled PD-L1-binding molecules, capable of capturing the entire extent of tumor heterogeneity, have been introduced as tracers to visualize PD-L1 expression in tumor-bearing animals and patients with NSCLC. Recent preclinical and clinical studies have demonstrated that these tracers can quantify PD-L1 expression in tumor cells in real-time, encouraging further development of molecular imaging for immunotherapy [14–19]. Owing to their relatively large size (150 kDa), antibody-based PD-L1-targeted tracers pose certain disadvantages, such as prolonged blood circulation, low tumor uptake, and limited tumor penetration. Nanobodies, single-domain antibodies derived from camels, offer a promising alternative owing to their lower molecular weight (12–15 kDa), facilitating deep tumor penetration, fast blood clearance, and easy modification and processing, increasing the potential for their use in constructing tracers for molecular imaging [10, 20–23]. In a previous study, we screened a PD-L1-targeted nanobody (NM-01) radiolabeled with ^99m^Tc as a single-photon emission computed tomography (SPECT)/CT tracer for the assessment of PD-L1 expression in NSCLC [10]. ^99m^Tc-labeled NM-01 exhibited an excellent safety profile, favorable imaging characteristics, and a significant correlation with PD-L1 IHC results in patients with NSCLC. In the present study, NM-01 was further conjugated with the chelator NODAGA and radiolabeled with ^68^Ga as a positron emission tomography (PET) tracer. Preclinical evaluation of ^68^Ga-labeled NM-01 (^68^Ga-NODAGA-NM-01) was conducted to validate its specific targeting ability *in vivo* in PD-L1-positive tumor-bearing mice. Furthermore, the safety, biodistribution, and tumor-targeting potential of ^68^Ga-NODAGA-NM-01 were assessed in a preliminary clinical study involving two patients with NSCLC.

## Methods

### Trial registration

^99m^Tc/^68^Ga Labeled Anti-PD-L1 sdAb in Assessment of PD-L1 Expression in NSCLC (NCT02978196; Registered February 15, 2018; https://www.clinicaltrials.gov/study/NCT02978196).

### Materials

The preparation and characterization of NM-01 are reported in our previous study [10]. NODAGA-NM-01 was synthesized by conjugating maleimide-NODAGA as described previously [24] and was kindly supplied by NanoMab (Shanghai, China). ^68^Ga was eluted from a ^68^Ge/^68^Ga generator (ITG, Baden-Württemberg, Germany) with 4 mL 0.05 M HCl solution. Phosphate-buffered saline (PBS), fetal bovine serum (FBS), and Dulbecco’s modified Eagle medium were purchased from Beijing BioDee Biotechnology Co., Ltd. (Beijing, China). All other chemicals and solvents were supplied by Sinopharm Chemical Reagent Co., Ltd. (Shanghai, China).

### Preparation and quality control of ^68^Ga-NODAGA-NM-01

NODAGA-NM-01 was labeled with ^68^Ga as described previously [25, 26]. In brief, a mixture of 150 μg NODAGA-NM-01, 1 mL ^68^GaCl_3_ (555–666 MBq), and 100 μL sodium acetate (1.0 M) was incubated at room temperature (20–25°C) for 10 min. The final product was analyzed using instant radio-layer chromatography (radio-TLC) and radio-high-performance liquid chromatography (radio-HPLC) according to the procedure described in our previous study [10]. Other quality control tests including visual checks, pH measurements, and endotoxin tests were performed. *In vitro* stabilities were confirmed by measuring the radiochemical purities (RCPs) of ^68^Ga-NODAGA-NM-01 in PBS at room temperature and in FBS at 37°C within 3 h.

### Cells and animal models

HCC827 and A549 cells were procured from Procell Life Science & Technology Co., Ltd. (Wuhan, China) and cultured as recommended by the supplier. Female BALB/c nude mice (4-week-old, 16–18 g) were sourced from the Shanghai Laboratory Animal Center of the Chinese Academy of Sciences (Shanghai, China). Tumor-bearing mouse models were established according to previously published protocols [27], wherein mice were subcutaneously inoculated with 100 μL cell suspension in their right-side flanks; the suspension contained 5 × 10^6^ cells in a 1:1 PBS and Matrigel mixture. Animal experiments were commenced when the tumor diameter reached 0.8–1.2 cm.

### Western blot analysis and saturation binding assays

PD-L1 expression levels in the A549 and HCC829 cells were verified by Western blotting. Total protein of tumor cells was extracted and quantified using a BCA protein assay kit according to a previously published procedure [27]. The blots were quantified using ImageJ software. HCC827 cells were inoculated into 24-well plates at a density of 2 × 10^5^ cells per well and incubated overnight. Different concentrations of ^68^Ga-NODAGA-NM-01 (0.01–70 nM) were added into the wells (*n* = 3) and incubated for 2 h at 37°C. The cells were washed twice with PBS and then lysed with pre-cooled NaOH (0.1M, 1 mL). Finally, the radioactivity was measured by a γ-counter (CAPINTEC, USA). Kd value was calculated using Graph pad Prism 8.0.

### Pharmacokinetics studies

Five 6-week-old healthy ICR mice were purchased from the Shanghai Laboratory Animal Center of the Chinese Academy of Sciences for evaluating the pharmacokinetic profile of ^68^Ga-NODAGA-NM-01. Each mouse was intravenously injected with ^68^Ga-NODAGA-NM-01 at a dose of 740 KBq in 200 μL saline solution. Blood samples (10 μL) were immediately collected and weighed after 1, 2, 5, 15, 30, 60, and 120 min, and the radioactivity was measured using a γ counter to calculate the percentage of injected dose per gram (%ID/g). Additionally, pharmacokinetic data were analyzed by DAS 2.0 (Shanghai, China) using a two-compartment model to calculate the half-life of ^68^Ga-NODAGA-NM-01 in blood.

### Micro-PET imaging study

PET imaging was performed using an Inveon small-animal PET scanner and the acquired images were reconstructed using Inveon Research software (Siemens Medical Solutions, Erlangen, Germany). Tumor-bearing mice were injected with 3.7–5.55 MBq ^68^Ga-NODAGA-NM-01 via the tail vein, equivalent to 10 µg NM-01 in 200 μL saline solution. For the blocking group, mice bearing HCC827 tumor xenografts were co-injected with 400 μg NM-01 and the same dose of ^68^Ga-NODAGA-NM-01. PET images were acquired 30 and 90 min after injection.

### Biodistribution

Mice with subcutaneous HCC827 or A549 xenografts were divided into two groups (five mice per group) to evaluate the biodistribution of ^68^Ga-NODAGA-NM-01 in the main organs and tumors. Each mouse was intravenously injected with ^68^Ga-NODAGA-NM-01 at a dose of 740 KBq in 200 µL saline solution. The mice were sacrificed 30 and 90 min after injection, and tumors and main organs, including the kidneys, liver, spleen, lungs, heart, and muscles, were collected. Each sample was immediately weighed and its radioactivity counted using a γ counter. The results are expressed as %ID/g.

### Preliminary clinical study

Two newly diagnosed patients with NSCLC between January and November 2019 were recruited and underwent PET/CT imaging and a follow-up after 1week. Patient inclusion criteria were age between 18 and 80 years with histopathologically diagnosed NSCLC, no prior lung cancer-related treatment, and an Eastern Cooperative Oncology Group Performance Score of 1 or less. Exclusion criteria included pregnancy or lactation, severe liver or kidney dysfunction, and previous chemotherapy, radiotherapy, or targeted therapy before PET/CT scans. PD-L1 expression in the available lesions was assessed through IHC, as described previously [10].

Patients were intravenously injected with ^68^Ga-NODAGA-NM-01 and asked to drink 300–500 mL water and empty their bladders before PET/CT imaging. ^68^Ga-NODAGA-NM-01 PET/CT scans were conducted at 1 h post-injection using a GE Discovery STE PET/CT scanner (GE Healthcare, Buckinghamshire, UK). The CT scans used a voltage of 140 kV, current of 150 mA, scanning layer thickness of 3 mm, and pitch of 1.2 mm. PET collections, lasting 3 min for each bed, were performed in two-dimensional mode. The ordered subset expectation maximization method was used for image reconstruction, and attenuation correction of the PET images was performed using CT projection scanning data processing to obtain whole-body fusion images of PET, CT, and PET/CT. Two experienced nuclear medicine physicians analyzed the images, delineated the regions of interest for the lesions, and calculated the maximum standardized uptake value (SUV_max_). In addition to the physiological uptake of the liver, spleen, kidney, and other parts of the image, when there was an abnormal concentration of local radioactivity or the uptake level was significantly higher than that of the surrounding normal tissues, the diagnosis was one of a primary tumor or metastasis after excluding the possibility of benign lesions, such as inflammation.

### Statistics

Data are presented as mean ± standard deviation. Statistical significance was evaluated using a one-way analysis of variance. A *p*-value of 0.05 was selected as the threshold of significance: * *p* < 0.05, ** *p* < 0.01, and *** *p* < 0.001.

## Results

### Preparation, quality control, and stability of ^68^Ga-NODAGA-NM-01

**Fig. 1:**
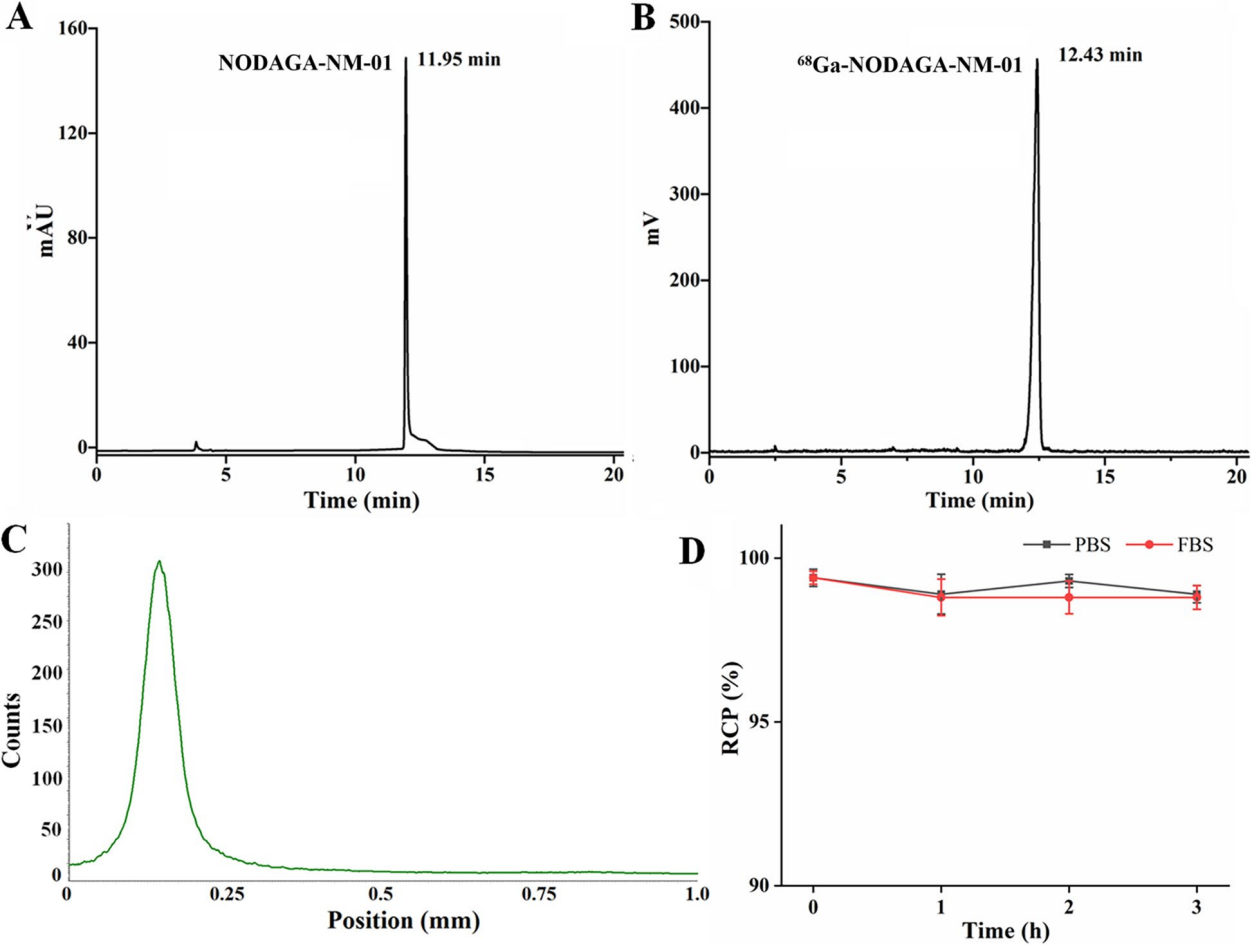
Characterization and stability of ^68^Ga-NODAGA-NM-01. **A**,** B **Radio-HPLC and (**C**) radio-TLC results of ^68^Ga-NODAGA-NM-01. **D ***In vitro* stability of ^68^Ga-NODAGA-NM-01 in phosphate-buffered saline (PBS) at room temperature and fetal bovine serum (FBS) at 37°C within 3 h

The NODAGA modified NM-01 was characterized by mass spectrometry and HPLC (Figs. S1 and 1A). The binding affinity of NM-01 to human PD-L1 (Kd = 0.8 nM) has been reported, which was not repeated in this study [28]. ^68^Ga-NODAGA-NM-01 could be readily prepared with high radiochemical yield (99.3 ± 0.5%) and specific activity (> 45Ci/g). ^68^Ga-NODAGA-NM-01 was characterized using radio-HPLC and radio-TLC. ^68^Ga-NODAGA-NM-01 displayed a single radioactive peak at 12.43 min (Fig. 1B), which is consistent with the retention time of NODAGA-NM-01 (11.95 min). The RCP of ^68^Ga-NODAGA-NM-01 was calculated to be greater than 98% without further purification, which could also be rapidly estimated using radio-TLC. ^68^Ga-NODAGA-NM-01 exhibited a retention factor of 0–0.2 (Fig. 1C), while ^68^GaCl_3_ had a retention factor of 0.8–1.0. Furthermore, the stability of ^68^Ga-NODAGA-NM-01 was analyzed in PBS at room temperature and in FBS at 37°C using radio-TLC. ^68^Ga-NODAGA-NM-01 was stable *in vitro*, with an RCP greater than 98% after 3 h (Fig. 1D).

### Western blot and binding affinity

**Fig. 2:**
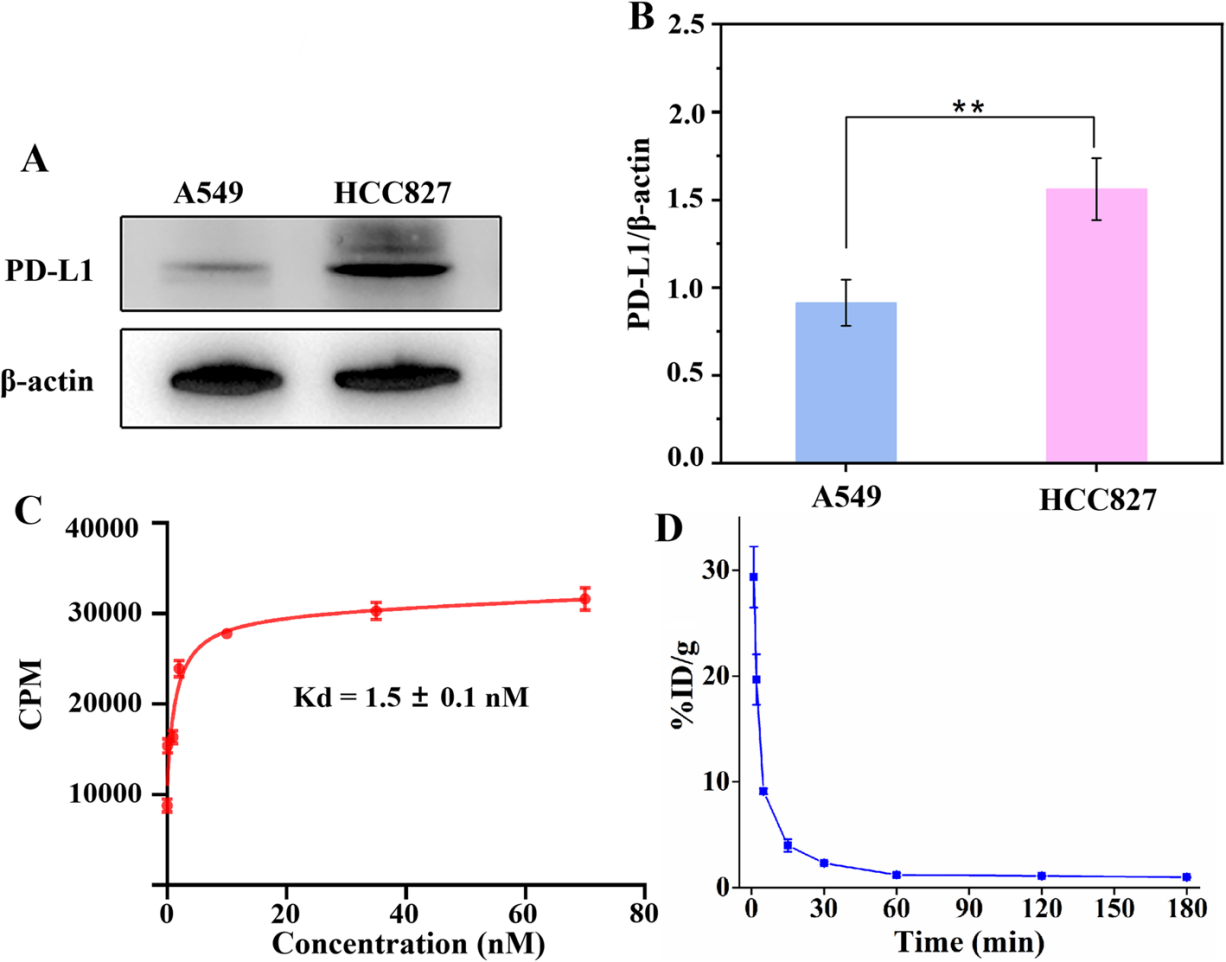
**A **Western blot results and (**B**) quantitative analysis of plectin expression in HCC827 and A549. **C **Saturation binding curve of ^68^Ga-NODAGA-NM-01 in HCC827 cells. **D **Blood clearance of ^68^Ga-NODAGA-NM-01 in normal ICR mice

PD-L1 expression levels in the selected tumor cells were examined using western blotting. As presented in Fig. 2A, HCC827 cells showed high PD-L1 expression levels, whereas low plectin expression were found in A549 cells. Quantitative analysis revealed a higher PD-L1 expression level in HCC827 cells than in A549 cells, further confirming the significance between these cells (Fig. 2B). To demonstrate the affinity of ^68^Ga-NODAGA-NM-01 to PD-L1, saturation binding assays were conducted in Hcc827 cells (Fig. 2C). With the increase of radioactivity concentration, the cell uptake of ^68^Ga-NODAGA-NM-01 gradually increased, and the dissociation constant (Kd) was calculated to be 1.5 ± 0.1 nM.

### Pharmacokinetics of ^68^Ga-NODAGA-NM-01

The radioactivity–time curve of ^68^Ga-NODAGA-NM-01 between 1 and 120 min post-injection is shown in Fig. 2D. At 1 min post-injection, blood radioactivity was 24.1 ± 3.9 %ID/g, which rapidly decreased to 7.8 ± 1.5 %ID/g 5 min post-injection. By 120 min post-injection, less than 2 %ID/g could be recovered from the blood pool. A two-phase exponential model was used to determine the biological blood half-life of ^68^Ga-NODAGA-NM-01. The elimination distribution phase half-life (t_1/2α_) and clear phase half-life (t_1/2β_) of ^68^Ga-NODAGA-NM-01 were approximately 1.02 and 35.66 min, respectively.

### Micro-PET/CT imaging and biodistribution

**Fig. 3:**
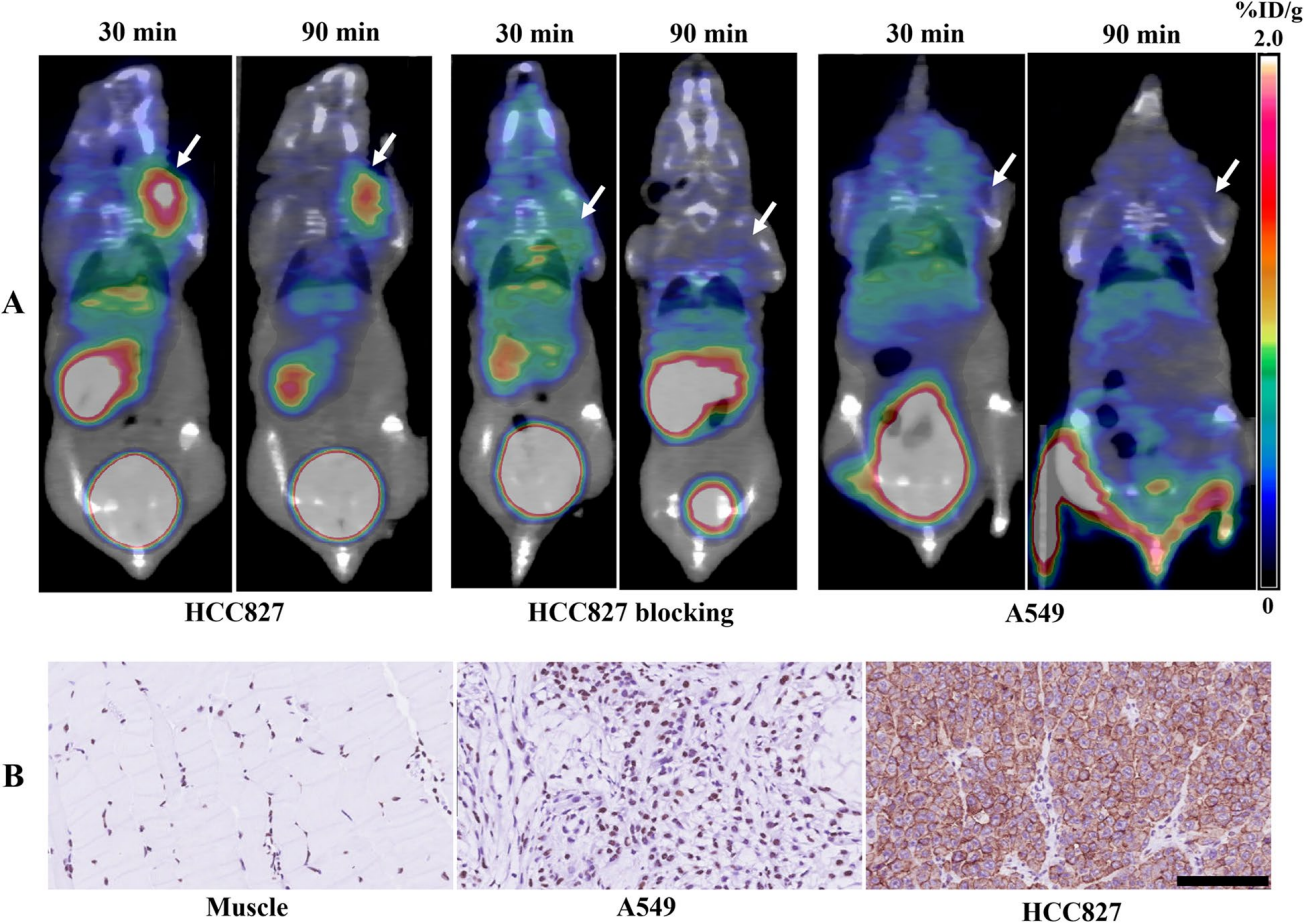
Uptake of ^68^Ga-NODAGA-NM-01 in tumors. **A **Micro-PET/CT images of ^68^Ga-NODAGA-NM-01 in PD-L1 positive HCC827 and PD-L1 negative A549 tumor-bearing mice. **B **Immunohistochemistry for PD-L1 in muscle, A549 tumors, and HCC827 tumors. Scale bar, 200 μm

Micro-PET/CT imaging was performed on PD-L1-positive HCC827 and PD-L1-negative A549 tumor-bearing mice. HCC827 tumors showed distinct high uptake of ^68^Ga-NODAGA-NM-01 30 and 90 min post-injection, whereas tumor uptake of ^68^Ga-NODAGA-NM-01 in the HCC827 blocking group and A549 tumors was negligible at both time points (Fig. 3A). High accumulation of ^68^Ga-NODAGA-NM-01 was observed in the kidneys and bladder, with low uptake in other major organs, such as the liver, spleen, heart, lung, and muscle. After micro-PET/CT imaging, IHC was performed to confirm PD-L1 levels in the tumors and analyze the correlation with tumor uptake of this probe; muscle tissues served as the negative control. IHC staining for PD-L1 in the same xenografts supported the PET/CT findings, revealing a higher uptake in tumors with higher PD-L1 levels (Fig. 3B).

**Fig. 4:**
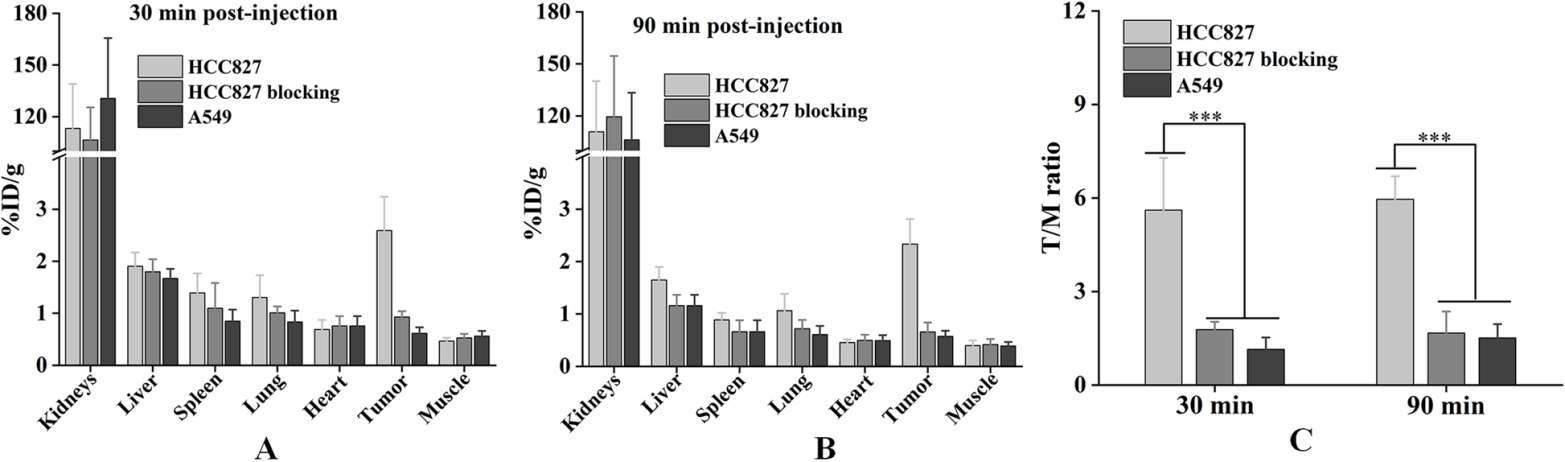
Biodistribution of ^68^Ga-NODAGA-NM-01 in tumors. Biodistribution in HCC827 and A549 tumor-bearing mice at (**A**) 30 and (**B**) 90 min post-injection, and (**C**) their tumor-to-muscle (T/M) ratios

To quantitatively analyze the radioactivity in the main organs and tumors in tumor-bearing mice, biodistribution experiments were performed at 30 and 90 min post-injection. The kidneys in the HCC827 tumor-bearing, HCC827 blocking, and A549 tumor-bearing mice had the highest uptake (113.11 ± 25.94 %ID/g, 106.38 ± 19.04 %ID/g, and 130.56 ± 35.06 %ID/g, respectively) 30 min post-injection, while the livers and other organs displayed low uptake, less than 2 %ID/g (Fig. 4A). Tumor uptake in HCC827 tumor-bearing mice was higher (2.59 ± 0.65 %ID/g) than in HCC827 blocking (0.93 ± 0.11 %ID/g) and A549 tumor-bearing mice (0.62 ± 0.11 %ID/g). The reduced tumor uptake in PD-L1-positive HCC827 after NM-01 blocking indicated the specificity of ^68^Ga-NODAGA-NM-01 *in vivo*. A slight decrease in uptake in the major organs and tumors was observed 90 min post-injection (Fig. 4B). Tumor uptake in HCC827 tumor-bearing, HCC827 blocking, and A549 tumor-bearing mice was 2.33 ± 0.48, 0.66 ± 0.18, and 0.57 ± 0.10 %ID/g, respectively. Accordingly, the tumor-to-muscle (T/M) ratio in HCC827 tumor-bearing mice (5.61 ± 1.68) was higher than that in HCC827 blocking (1.78 ± 0.25) and A549 tumor-bearing mice (1.15 ± 0.38) 30 min post-injection (Fig. 4C), and T/M ratios 90 min post-injection increased to 5.96 ± 0.74, 1.67 ± 0.70, and 1.51 ± 0.44, respectively.

### Preliminary clinical study of ^68^Ga-NODAGA-NM-01 PET/CT imaging

Two male patients (aged 77 and 69 years) with histopathologically confirmed NSCLC (one squamous cell carcinoma and one adenocarcinoma) were enrolled for a preliminary clinical study. One patient tested positive for PD-L1 expression, whereas the other tested negative. The administered doses of ^68^Ga-NODAGA-NM-01 were 82 and 107 MBq, respectively, corresponding to 75 μg of NODAGA-NM-01. No drug-related adverse effects were observed during the 7-day follow-up period. There were no significant changes in vital clinical signs, blood and urine laboratory test results, and other clinical parameters such as heart rate, respiratory rate, body temperature, and blood pressure.

**Fig. 5:**
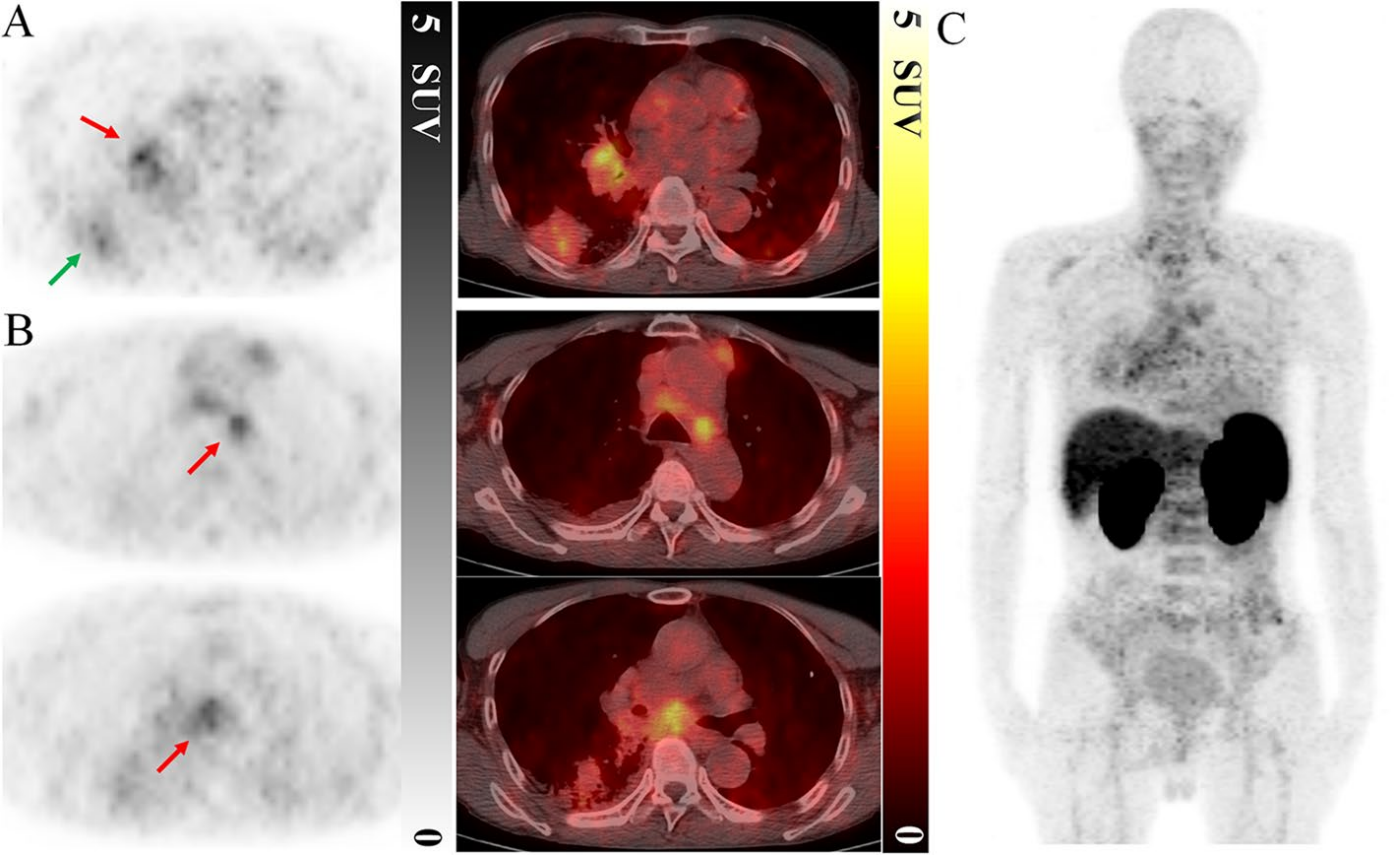
^68^Ga-NODAGA-NM-01 PET/CT of patient 1. Different uptake in the (**A**) right lung lower lobe primary lesion (green arrow), right hilar lymph node (red arrow), and (**B**) mediastinal lymph metastases. The SUV_max_ of primary lesion and metastases were 3.3, 4.4, and 3.6, respectively. **C **Representative maximum-intensity-projection image of patient 1

**Fig. 6:**
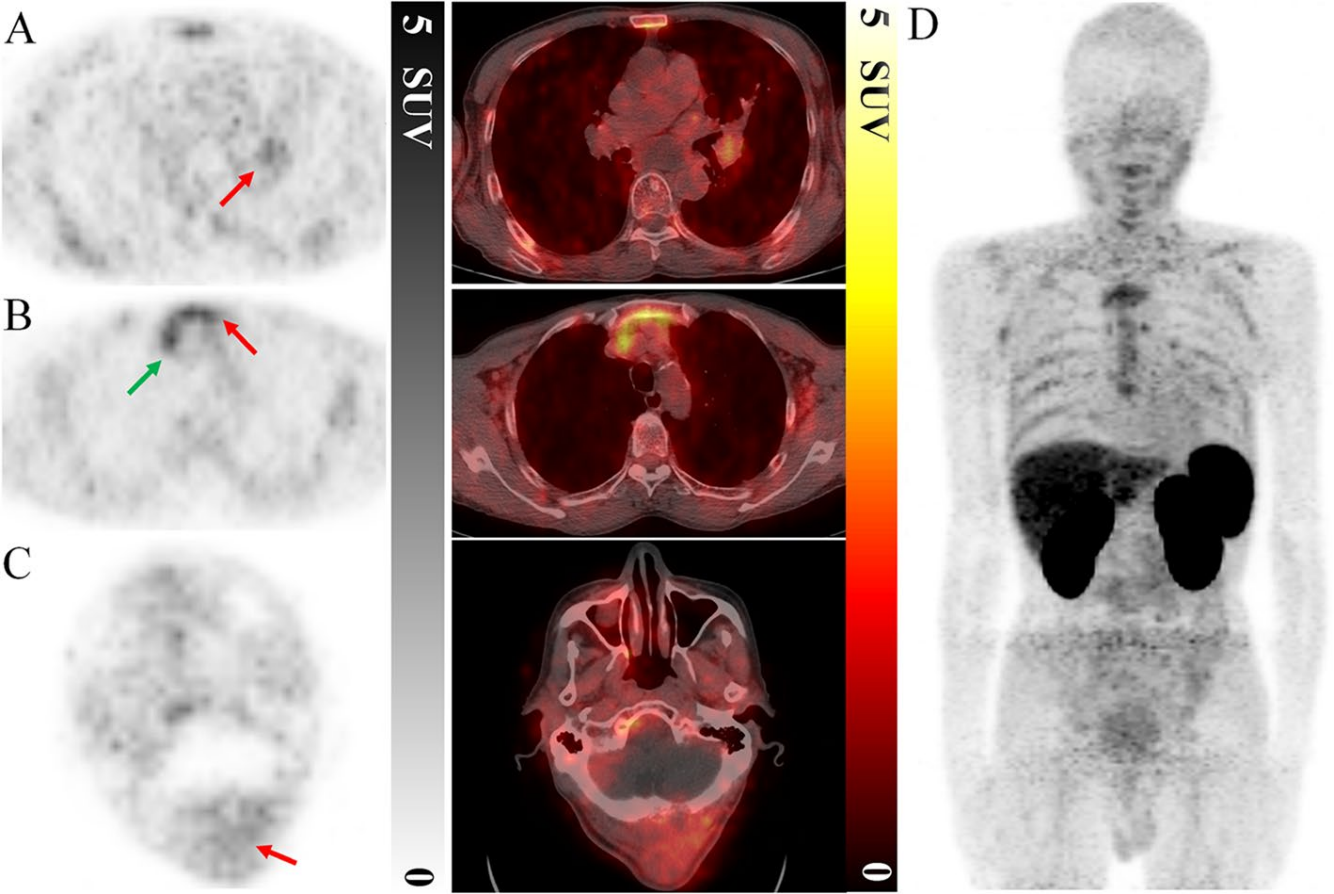
^68^Ga-NODAGA-NM-01 PET/CT of patient 2. Mild uptake in the (**A**) left hilum primary lesion with SUV_max_ of 1.7, while heterogeneous uptake in the (**B**) mediastinal lymph node with SUV_max_ of 2.4 (green arrow), sternum metastasis with SUV_max_ of 2.8 (red arrow), and (**C**) occipital metastasis with SUV_max_ of 1.5, respectively. **D **Representative maximum-intensity-projection image of patient 2

Following PET/CT imaging, two experienced nuclear medicine physicians independently conducted visual interpretation and quantitative analysis of all lesions. The PET/CT images of the two patients with NSCLC are shown in Figs. 5 and 6. Biodistribution of ^68^Ga-NODAGA-NM-01 was as expected with main radioactivity in the liver, spleen, and kidneys. Low accumulation was found in the lungs, bone marrow and muscle. In terms of tumor uptake, patient 1 displayed moderate uptake of ^68^Ga-NODAGA-NM-01 in the primary lesion and metastases. The SUV_max_ of the right lower lobe primary lesion and right hilar lymph node were 3.3 and 4.4, respectively (Fig. 5A), and the SUV_max_ of the mediastinal lymph nodes were 3.5 and 3.6 (Fig. 5B). IHC staining confirmed PD-L1 positivity in the primary tumor. Physiologically, ^68^Ga-NODAGA-NM-01 mainly accumulated in the kidneys, liver, and spleen, with a relatively low uptake in the muscle and bone marrow (Fig. 5C). In patient 2, the primary lesion exhibited low uptake of ^68^Ga-NODAGA-NM-01 with an SUV_max_ of 1.7 (Fig. 6A), consistent with negative PD-L1 expression as confirmed by IHC. The left hilar lymph node and bone metastases had mild levels of tracer uptake with SUV_max_ of 2.4 and 2.8 (Fig. 6B), while a low SUV_max_ of 1.5 in the occipital metastasis (Fig. 6C) and background levels in the bone marrow and muscle were observed (Fig. 6D). This suggests varying PD-L1 expression between primary lesions and metastases.

## Discussion

PD-1/PD-L1 blockade therapy has emerged as a promising approach for managing NSCLC, although its effectiveness is limited to a subset of patients. Many radiolabeled PD-L1-targeted imaging ligands, such as antibodies, nanobodies and peptides, have been evaluated in preclinical and clinical studies to dynamically and non-invasively assess PD-L1 status, potentially extending the benefits of PD-1/PD-L1 blockade therapy to a larger population [14, 17–19]. Despite the successful use of monoclonal antibodies, such as ^89^Zr-atezolizumab and ^89^Zr-durvalumab, for *in vivo* tumor imaging, their applications are still restricted by their large molecular size, which prevent tumor penetration and plasma clearance. By contrast, nanobody- and peptide-based tracers have gained significance due to their smaller molecular size and excellent targeting properties, allowing rapid blood clearance and a high tumor-to-background ratio. In our previous study, we developed a nanobody, NM-01, labeled with ^99m^Tc as a SPECT probe for non-invasive assessment of PD-L1 expression [10]. We demonstrated its safety, tolerable radiation dose, favorable biodistribution, and image characteristics correlating with PD-L1 IHC results. Based on these findings, in the present study, we synthesized ^68^Ga-NODAGA-NM-01 and assessed its potential for PET imaging of PD-L1 expression in NSCLC.

The radiolabeling strategy for NM-01 is site-specific and simple. ^68^Ga-NODAGA-NM-01 was prepared within 30 min with a high RCP and good stability *in vitro*. Prior to the preliminary clinical study, we validated the imaging performance of ^68^Ga-NODAGA-NM-01 *in vivo* using tumor-bearing mice. High uptake of ^68^Ga-NODAGA-NM-01 in PD-L1-positive tumors (HCC827), but low accumulation in the blocking group and PD-L1-negative tumors (A549), confirmed the specificity of the tracer for PD-L1. The tumor retention of radioactivity remained stable but was rapidly cleared from the blood, resulting in good-quality PET images with high T/M ratios as early as 0.5 h post-injection. This was attributed to its hydrophilicity and small molecular size. Similar to other radiolabeled PD-L1-targeted nanobodies, the radioactivity of ^68^Ga-NODAGA-NM-01 mainly in the liver, spleen, and kidneys with low background levels in the lungs, bone marrow and muscle. The tumor uptake of ^68^Ga-NODAGA-NM-01 was 2.59 ± 0.65 %ID/g at 0.5 h post-injection, which was close to other reported nanobody-based tracers, such as ^68^Ga-NOTA-Nb109 and ^68^Ga-NOTA-RW102 [17, 29]. Notably, ^68^Ga-NODAGA-NM-01 showed lower tumor uptake than some reported nanobodies and peptides, such as ^68^Ga-THP-APN09 and ^68^Ga-NOTA-WL12 [30, 31]. This was probably related to the high PD-L1 levels in the used mouse xenograft models constructed by gene-transfected tumor cells. Meanwhile, several peptide-based agents also demonstrated high tumor uptake in natural high PD-L1-expressing tumor models, providing support for clinical studies and future development of immunoimaging technology [18, 32, 33].

A preliminary clinical study of ^68^Ga-NODAGA-NM-01 was conducted in patients with NSCLC with varying PD-L1 expression status (PD-L1 positive and negative). We demonstrated that ^68^Ga-NODAGA-NM-01 PET/CT is a safe procedure with no adverse events. The PET/CT images of the primary lesions were carefully evaluated and compared with PD-L1 IHC results. The primary lesion of the PD-L1-positive patient was clearly visualized with a low background signal 1 h post-injection, whereas the primary lesion of the PD-L1-negative patient showed low uptake, consistent with their IHC results and SUV_max_. However, probably owing to the heterogeneity of PD-L1 expression, metastases of both PD-L1-positive and -negative patients showed distinct accumulation of radioactivity. This hypothesis requires further investigation. Similar to ^99m^Tc-NM-01 and other nanobody-based PD-L1 tracers, ^68^Ga-NODAGA-NM-01 exhibited the highest radioactivity in the kidneys [34–36]. Therefore, efforts must be made to reduce high kidney retention [37].

Although our data demonstrated that the ability of ^68^Ga-NODAGA-NM-01 to reflect PD-L1 expression in primary and metastatic lesions of patients with NSCLC, the present study has potential limitations. To further verify the safety and efficiency of this tracer, a relatively large cohort is needed. Bone and lymph node metastases that were positive for PD-L1 expression on ^68^Ga-NODAGA-NM-01 PET/CT were not examined pathologically, opening the possibility of false-positive results. In addition, without tracking and follow-up, we were unable to investigate the potential of ^68^Ga-NODAGA-NM-01 PET/CT imaging for assessing the prognosis of patients undergoing or not having received immunotherapy. Future studies with ^68^Ga-NODAGA-NM-01 PET/CT imaging may address these issues, improving our understanding of its potential application for guiding NSCLC treatment.

## Conclusions

A ^68^Ga-labeled PD-L1 targeted nanobody was successfully prepared using a simple method under mild conditions. The prepared ^68^Ga-NODAGA-NM-01 had a high RCP, good stability *in vitro*, rapid blood clearance, and specific accumulation in PD-L1-positive tumors *in vivo*. A preliminary clinical study showed that ^68^Ga-NODAGA-NM-01 PET/CT imaging was a safe procedure with no adverse events in two patients with NSCLC that could reflect PD-L1 expression in primary and metastatic lesions. However, further validation in a larger patient cohort is warranted to substantiate these findings.

## Supplementary Information

Supplementary Material 1.

## Data Availability

No datasets were generated or analysed during the current study.

## Abbreviations

FBS: Fetal bovine serum
IHC: Immunohistochemistry
NSCLC: Non-small-cell lung cancer
PBS: Phosphate-buffered saline
PD-1: Programmed cell death 1
PD-L1: Programmed death ligand-1
PET: Positron emission tomography
Radio-HPLC: Radio-high-performance liquid chromatography
Radio-TLC: Radio-layer chromatography
RCP: Radiochemical purity
SPECT: Single-photon emission computed tomography
SUV_max_: Maximum standardized uptake value
T/M: Tumor-to-muscle
t_1/2α_: Elimination distribution phase half-life
t_1/2β_: Clear phase half-life
^68^Ga-NODAGA-NM-01: ^68^Ga-labeled NM-01
%ID/g: Percentage of injected dose per gram
