# Supplementary material for: Preclinical evaluation and preliminary clinical study of 68Ga-NODAGA-NM-01 for PET imaging of PD-L1 expression
Source: Cancer Imaging. 2025 Jan 27;25:6. doi: 10.1186/s40644-025-00826-8 (PMC11771120; doi:10.1186/s40644-025-00826-8)
Supplement: Supplementary file 1 — Supplementary Material 1. [file 40644_2025_826_MOESM1_ESM.docx]

**Supporting information**

**
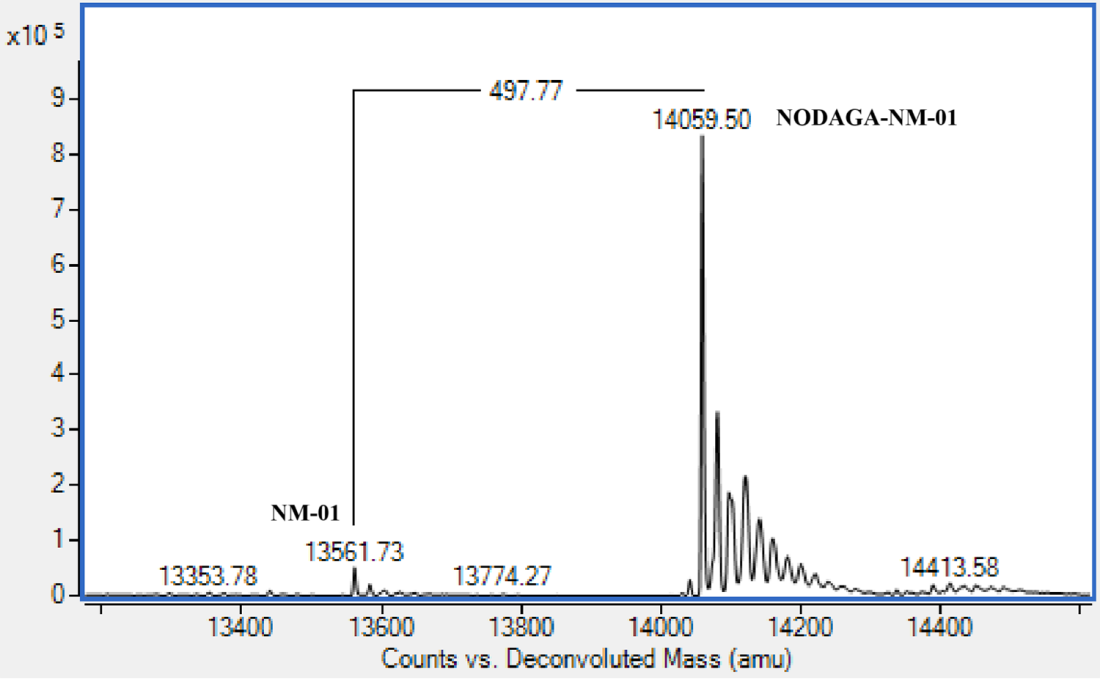
**

**Figure S1.** The mass spectrometry of NODAGA-NM-01 (supplied by the manufacturer).
